# High-Intensity Laser Therapy Versus Extracorporeal Shockwave Therapy for Plantar Fasciitis: A Systematic Review and Meta-Analysis

**DOI:** 10.3390/bioengineering13010090

**Published:** 2026-01-13

**Authors:** Pei-Ching Wu, Dung-Huan Liu, Yang-Shao Cheng, Chih-Sheng Lin, Fu-An Yang

**Affiliations:** 1 Department of Chinese Medicine, China Medical University Hospital, Taichung City 404327, Taiwan; 036619@tool.caaumed.org.tw; 2 College of Chinese Medicine, China Medical University, Taichung City 404333, Taiwan; 3 Graduate Degree Program of Biomedical Science and Engineering, National Yang Ming Chiao Tung University, Hsinchu City 300193, Taiwan; 035345@tool.caaumed.org.tw (D.-H.L.); lincs@mail.nctu.edu.tw (C.-S.L.); 4 Department of Physical Medicine and Rehabilitation, Taichung Municipal Geriatric Rehabilitation General Hospital, China Medical University, Taichung City 406004, Taiwan; 5 Department of Physical Medicine and Rehabilitation, China Medical University Hospital, Taichung City 404327, Taiwan; hsnu128439@gmail.com; 6 Department of Physical Therapy, Graduate Institute of Rehabilitation Science, China Medical University, Taichung City 406040, Taiwan; 7 Department of Education, China Medical University, Taichung City 404333, Taiwan; 8 Department of Biological Science and Technology, National Yang Ming Chiao Tung University, Hsinchu City 300193, Taiwan; 9 Center for Intelligent Drug Systems and Smart Bio-Devices (IDS2B), National Yang Ming Chiao Tung University, Hsinchu City 300193, Taiwan; 10 Department of Physical Medicine and Rehabilitation, Far Eastern Memorial Hospital, New Taipei City 220216, Taiwan; 11 Department of Internal Medicine, China Medical University Hospital, Taichung City 404327, Taiwan; 12 School of Medicine, College of Medicine, Taipei Medical University, Taipei 110301, Taiwan

**Keywords:** high-intensity laser therapy, extracorporeal shockwave therapy, plantar fasciitis, systematic review, meta-analysis

## Abstract

Background: Plantar fasciitis is a prevalent musculoskeletal disease characterized by heel pain and functional impairment. Both high-intensity laser therapy (HILT) and extracorporeal shockwave therapy (ESWT) have demonstrated efficacy in managing plantar fasciitis; however, their relative effectiveness remains unclear. Purpose: This systematic review and meta-analysis aimed to compare the effects of HILT and ESWT for treating plantar fasciitis. Methods: A comprehensive literature search of PubMed, the Cochrane Library, EMBASE, and Scopus was conducted from inception to 13 July 2025 to identify randomized controlled trials (RCTs) investigating both interventions. Two reviewers independently extracted data and assessed the methodological quality of the trials using the Physiotherapy Evidence Database (PEDro) scale. The certainty of evidence was evaluated using the Grading of Recommendations Assessment, Development and Evaluation (GRADE) approach. The primary outcomes of this study were pain intensity and foot function. The visual analog scale (VAS) was used for pain assessment. Foot function was evaluated by the total scores of the Foot Function Index (FFI) and American Orthopedic Foot & Ankle Society Scale (AOFAS) and the activities of daily living (ADL) subscale scores of the Foot and Ankle Ability Measure (FAAM). Outcomes were assessed at the end of treatment and during short-, medium-, and long-term follow-ups. The meta-analysis utilized standardized mean differences (SMDs), assessed heterogeneity using the I^2^ test, applied the inverse variance method for pooling continuous variables, and employed a random-effects model because of the variable study methods used across the included articles. Results with *p* < 0.05 were considered statistically significant. The I^2^ test was used to objectively measure statistical heterogeneity, with I^2^ ≥ 50% indicating significant heterogeneity. Results: Five RCTs met the inclusion criteria, with methodological quality scores ranging from 6 to 7 on the 10-point PEDro scale. In total, 120 participants received HILT and 116 received ESWT. Regarding pain intensity (VAS), no statistically significant differences were detected between HILT and ESWT at any time point, including short-term morning pain (SMD = −0.11, 95% CI −0.42 to 0.19, *p* = 0.40), resting pain (SMD = 0.01, 95% CI −0.48 to 0.49, *p* = 0.05), and activity pain (SMD = −0.08, 95% CI −0.41 to 0.26, *p* = 0.89), as well as medium-term morning, resting, and activity pain (all *p* > 0.05). For foot function (FFI), the pooled analysis of all studies showed no significant short-term difference (SMD = 0.37, 95% CI −0.22 to 0.95, *p* = 0.01; I^2^ = 73%); however, a subsequent sensitivity analysis, which excluded one studyreduced heterogeneity to 0% and revealed a significant short-term advantage of ESWT (SMD = 0.64, 95% CI 0.32 to 0.95, *p* < 0.01). Medium-term FFI also favored ESWT (SMD = 0.53, 95% CI 0.14 to 0.92, *p* < 0.01). Overall, the certainty of evidence ranged from moderate to low, mainly due to risk of bias and heterogeneity, as assessed by the GRADE approach. Conclusions: While the pooled results suggested a trend toward greater functional improvement with ESWT than with HILT in the short- and medium-term, the effect sizes were small. No significant between-group differences were observed in pain-related outcomes. Given the limited number of available trials and variability in treatment protocols, current evidence remains insufficient to draw definitive conclusions about the comparative efficacy of ESWT and HILT. Further high-quality, large-scale randomized controlled trials with standardized methodologies are needed to better inform clinical decision-making.

## 1. Introduction

Plantar fasciitis (PF) is a common foot problem that accounts for 15% of all foot diseases, is most prevalent in individuals aged 40 to 60 years, and affects 17.4% of runners [1]. The symptoms of PF are characterized by chronic medial heel pain exacerbated by weight bearing and persisting after resting. Typically, symptoms can last more than a year even after treatment [2], resulting in negative impacts on patients’ quality of life and social economy. The pathology of PF is still unknown. Despite its name, previous studies have reported secondary degenerative changes caused by repetitive trauma in the PF without inflammation [3,4]. Various conservative treatments have been proposed to manage the symptoms of PF, including steroid injection, platelet-rich plasma injection, gold-induced cytokine injection therapy, extracorporeal shockwave therapy (ESWT), high-intensity laser therapy (HILT), and mechanical treatments, including insoles, orthoses, night splints, and specialized shoes [2,5,6,7]. The 2023 Heel Pain–Plantar Fasciitis clinical practice guideline outlines a stepwise, evidence-based treatment pathway that prioritizes core conservative care (e.g., manual therapy, stretching, taping) and then adds adjunct options (e.g., night splints and selected physical agents such as low-level laser therapy (LLLT) for short-term pain relief) based on patient response [8]. However, the most effective management method has not been determined.

Surgical approaches are considered when conservative measures fail. Minimally invasive techniques such as percutaneous plantar fascia release offer a reduced risk of skin complications, nerve injury, and prolonged recovery [9], whereas endoscopic plantar fascia release has shown durable outcomes in long-term follow-up, improving functional scores with minimal complications [10].

ESWT should be considered before surgical treatment in patients with refractory chronic PF who fail to respond to other conservative treatments [11]. It appears to have better long-term outcomes than corticosteroid injections and other management methods [12]. ESWT delivers either focused or radial acoustic waves to stimulate biological repair. Focused ESWT penetrates deeper and concentrates energy in a small focal zone, making it suitable for targeting deep lesions, whereas radial ESWT disperses lower energy waves over a broader area, generally treating more superficial soft tissue structures. Both forms are widely used in PF management, yet their relative efficacy remains debated [2,13,14]. ESWT relieves pain in the PF by destroying unmyelinated sensory fibers and stimulating neovascularization and collagen synthesis in degenerative tissues [14]. Approximately 20% of patients who undergo ESWT experience side effects such as temporary skin redness and ecchymosis [15]. The 2023 evidence-based practice guidelines summarizes ESWT as a noninvasive modality using direct mechanical forces with meta-analytic support for medium- to long-term pain reduction, and notes that adding ESWT to a multimodal program may yield small-to-moderate short- to medium-term gains in pain and function [8].

HILT is a form of photobiomodulation therapy that utilizes pulsed Nd:YAG lasers with higher peak power and energy density than LLLT. Photobiomodulation refers to the light-induced modulation of cellular processes, which enhances tissue repair, modulates inflammation, and provides analgesia without inducing thermal damage. HILT typically employs wavelengths of approximately 1064 nm and peak powers ranging from 1 to 75 watts, enabling deeper penetration and the induction of photothermal and photomechanical effects [16]. In contrast, LLLT generally uses lower power (<0.5 W) and shorter wavelengths (e.g., 830–850 nm), theoretically limiting its therapeutic reach to more superficial tissues [16,17]. These mechanistic and dosimetric differences may underlie the variability in clinical outcomes observed between the two modalities. The guideline gives a Grade B recommendation for LLLT as part of rehabilitation to reduce short-term pain in acute or chronic plantar fasciitis, while HILT is not graded and is mentioned only as a comparator in the LLLT evidence update, where pooled short-term effects show no significant difference between LLLT and HILT [8]. HILT is a new, noninvasive and painless treatment option, and its anti-inflammatory, antiedema, and analgesic effects have been reported [18,19]. The application of HILT in many musculoskeletal diseases, including PF, has been proven effective, with favorable results [20,21].

Both ESWT and HILT have been reported to have pain-relieving effects on PF. A systematic review by Charles et al. [22] reported moderate-to-high certainty of evidence from thirteen randomized controlled trials. They found that ESWT produces a large short-term improvement in function and a large reduction in pain across short-, mid-, and long-term follow-up. Similarly, another systematic review conducted by Yadav et al. [23] summarized evidence from multiple trials demonstrating that HILT significantly reduces pain and improves function, with some studies suggesting that laser therapy may provide superior short-term pain reduction compared with ESWT. Despite strong evidence for each treatment individually, no systematic review to date has directly compared the efficacy of ESWT and HILT in plantar fasciitis. This study was therefore designed to address this gap by directly comparing the clinical efficacy of ESWT and HILT in the treatment of PF.

## 2. Methods

This review was performed in accordance with the recommendations of the Cochrane Handbook for Systematic Reviews of Interventions [24] and is reported following the guidelines of the Preferred Reporting Items for Systematic Reviews and Meta-Analyses [25]. This systematic review was registered in the International Prospective Register of Systematic Reviews database under the number CRD420251025231 on 3 April 2025.

### 2.1. Search Strategy and Eligibility Criteria

Randomized controlled trials (RCTs), including pilot and crossover studies, were included. Eligibility was determined using the Patient, Intervention, Comparison, Outcome, Study designs (PICOS) model: Participants were individuals diagnosed with plantar fasciitis (PF) of any severity; the intervention of interest was high-intensity laser therapy (HILT); the comparison group received extracorporeal shockwave therapy (ESWT) as the control treatment; and the outcomes focused on relevant clinical measures, specifically pain and foot function. The study design was a Systematic Review and Meta-analysis. Articles that were protocols, non-peer-reviewed articles, conference papers, or letters to the editor were excluded. Moreover, crossover studies that did not include a washout period were excluded. No language restriction was applied in our search strategy.

The PubMed, EMBASE, Scopus and Cochrane Library electronic databases were searched. In our search strategy, terms related to PF, HILT and ESWT and equivalent terms (the search strategies are presented in the Appendix A) were included. If available, RCTs were identified using the refined search function of the databases. Additional articles were identified by manually searching the reference lists of relevant articles. The databases were searched from their inception to 13 July 2025. Two reviewers independently evaluated the eligibility of all titles and abstracts, and disagreements were resolved through discussion. If necessary, a third reviewer was involved. The full texts of the remaining articles were subsequently screened to determine the eligibility of the articles.

### 2.2. Relevant Outcomes

Two authors extracted data from each study by using a structured form, and the characteristics of all eligible studies are summarized in a table. The following data were extracted: (1) basic information of the qualifying studies (first author and publication date); (2) demographic, clinical, and treatment characteristics (e.g., number and mean age of patients in the control and treatment groups); (3) treatment protocol, duration, and follow-up period; and (4) outcome measures. The means and standard deviations of the outcome measures posttreatment for the experimental and control groups were extracted. If crucial data could not be extracted from an article, we sent an email to the corresponding author requesting the data. The primary outcomes of this study were pain intensity and foot function. The visual analog scale (VAS) for pain is a 10 cm (100 mm) horizontal line anchored by “no pain” (0) and “the most severe/unbearable pain you ever felt in your lifetime” (10), on which patients mark their pain intensity for a corresponding score [26]. The Foot Function Index (FFI) is a versatile tool used across all ages to measure the impact of foot conditions (congenital, acute, chronic diseases, and injuries) on pain, disability, and activity restriction, with higher scores indicating greater disability. It is also used to evaluate treatment effectiveness for surgical interventions or orthoses [27]. The American Orthopedic Foot and Ankle Society (AOFAS) score is a rearfoot questionnaire designed to assess disability, consisting of nine questions assessing pain, function, and foot alignment [28]. Finally, the Foot and Ankle Ability Measure (FAAM) is a 29-item self-report questionnaire for assessing disability. It is divided into a 21-item activities of daily living (ADL) subscale and an 8-item sport subscale, with lower scores indicating greater disability [29]. Each question uses a 5-point Likert scale, with subscale scores ranging from 0 to 100 (worst to best outcome) [29]. The foot function comparison in this study was evaluated by the total scores of the FFI and AOFAS and the ADL subscale scores of the FAAM. Follow-up periods of less than 1 month were defined as short-term, periods of 1 to 3 months were defined as medium-term, and periods of more than 3 months were defined as long-term.

### 2.3. Risk of Bias Assessment

The quality of the included studies was assessed using the Physiotherapy Evidence Database (PEDro) scale, which is widely used for evaluating the risk of bias in RCTs [30]. The PEDro scale scores provided by the two assessors were compared, and differences were resolved through discussion with a third researcher. The ratings of PEDro scale items 2–11 are summed to obtain a total PEDro scale score ranging between 0 and 10 [30]. Scores of <4 are considered “poor,” scores of 4–5 are considered “fair,” scores of 6–8 are considered “good,” and scores of 9–10 are considered “excellent” [30]. All the articles were included in this review irrespective of their PEDro score.

### 2.4. Statistical Analysis

Statistical analyses were performed using RevMan 5.4 software (Version 5.4. Copenhagen, Denmark), which is provided by the Cochrane Collaboration (https://training.cochrane.org/online-learning/core-software-cochrane-reviews/revman/revman-5-download (Accessed on 5 April 2025). Continuous postintervention data were extracted. For studies not reporting standard deviations, the authors were contacted for raw data, or if unavailable, the data were estimated by calculating correlation coefficients in accordance with the instructions provided in the Cochrane Handbook for Systematic Reviews of Interventions [24]. If studies included more than two intervention groups, we combined relevant groups to create a single comparison or selected the most appropriate control group in line with the Cochrane guidelines. The results with *p* < 0.05 were considered statistically significant. The I^2^ test was used to objectively measure statistical heterogeneity, with I^2^ ≥ 50% indicating significant heterogeneity [31]. A random-effects model was consistently used in this meta-analysis to account for the expected clinical and methodological diversity among the included studies, acknowledging that different study methods contribute to variation beyond chance. Continuous variables were pooled using standardized mean differences (SMDs) calculated with the inverse variance method and presented with 95% confidence intervals (CIs). We opted for SMDs over mean differences (MDs) because the studies used different outcome measurement scales for pain and foot function, making SMDs appropriate for combining results from varying instruments. SMDs, calculated using Cohen’s d, were employed to measure the probable clinical meaningfulness of the relationships between variables in a population. An SMD of <0.2 indicated a clinically meaningless effect; an SMD of 0.2–0.5 indicated a small effect; an SMD of 0.5–0.8 indicated a moderate effect; and an SMD of >0.8 indicated a large effect [32]. A funnel plot was constructed to examine publication bias if the number of studies included in each analysis was greater than 10. Sensitivity analyses were performed by excluding studies at high risk of bias (PEDro score < 5) to assess the robustness of our findings.

In this study, the FFI and AOFAS scale were used, where higher scores for FFI indicate greater disability, while higher scores for AOFAS and FAAM represent better function. To ensure consistency for the meta-analysis, we followed the recommendations in the Cochrane Handbook by multiplying the mean values of scales with opposite directions (AOFAS and FAAM) by −1. This ensured that for all pooled functional outcomes, a higher SMD consistently indicated a better outcome for the ESWT group compared to the HILT group.

### 2.5. Confidence of Evidence

The Grading of Recommendations, Assessment, Development, and Evaluation (GRADE) approach was used to measure the quality of evidence as confidence in effect estimates [33]. This method examines the quality of the publication on the basis of the study design (randomized trials vs. nonrandomized design) and five key domains: risk of bias, inconsistency, imprecision, indirectness, and publication bias. Specifically, risk of bias was assessed using the PEDro scale; inconsistency was evaluated by examining statistical heterogeneity (I^2^ statistic) and overlapping confidence intervals; imprecision was judged on the basis of the width of the confidence intervals and optimal information size; indirectness considered differences in population, intervention, comparison, or outcomes from the review question; and publication bias was assessed visually using funnel plots when applicable. The size and trend of the effect were also considered [33].

## 3. Results

### 3.1. Searching Process

**Figure 1:**
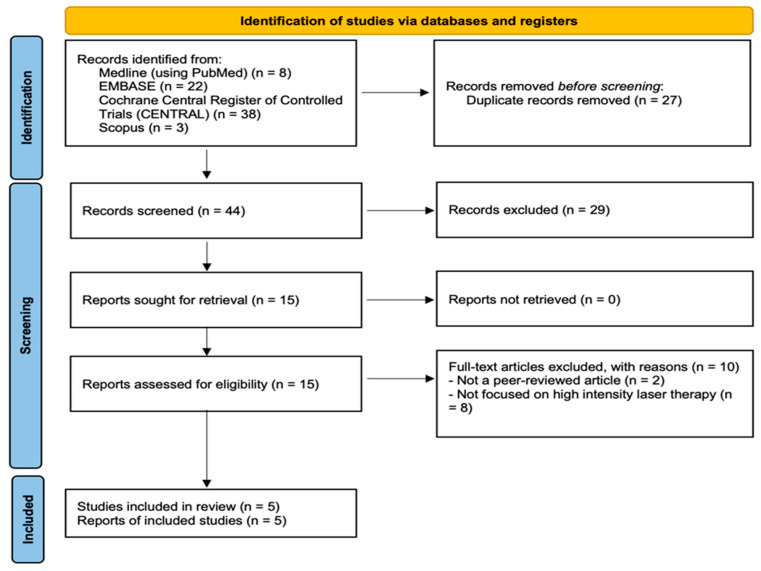
Article selection process.

By using the search terms reported in the Appendix A, 71 studies (8 studies from PubMed, 22 studies from EMBASE, 3 studies from Scopus and 38 from the Cochrane Library) were initially retrieved. Among these, 27 duplicates were excluded using EndNote 20 (version 20; Clarivate, Philadelphia, PA, USA) [34]. Furthermore, 29 studies that did not meet the inclusion criteria were excluded after their titles and abstracts were screened. The authors screened the full texts of the remaining 15 articles and determined that 10 studies did not fulfill our inclusion and exclusion criteria. Finally, 5 studies were included in our study [6,35,36,37,38]. Figure 1 presents the flowchart of article selection.

### 3.2. Study Characteristics

The selected studies included 120 and 116 participants in the intervention and control groups, respectively. All of the selected studies were RCTs [6,35,36,37,38]. Five studies reported pain outcomes [6,35,36,37,38], 1 reported foot and ankle function [36], 2 reported the Foot Function Index [35,37], and 1 reported the AOFAS score [38]. Table 1 lists the characteristics of the included studies. Table 2 and Table 3 list the parameters of the HILT and ESWT protocols.

### 3.3. Results of Risk of Bias Assessment

Two reviewers independently evaluated the quality of the included RCTs by using a PEDro scale [30]. Four studies received PEDro scale scores of 7 [6,35,36,37], and 1 received a score of 6 [38]. All of the studies were considered “good” quality [6,35,36,37,38]. Table 4 summarizes the results of the risk of bias assessment.

### 3.4. Short-Term Morning Pain

**Figure 2:**
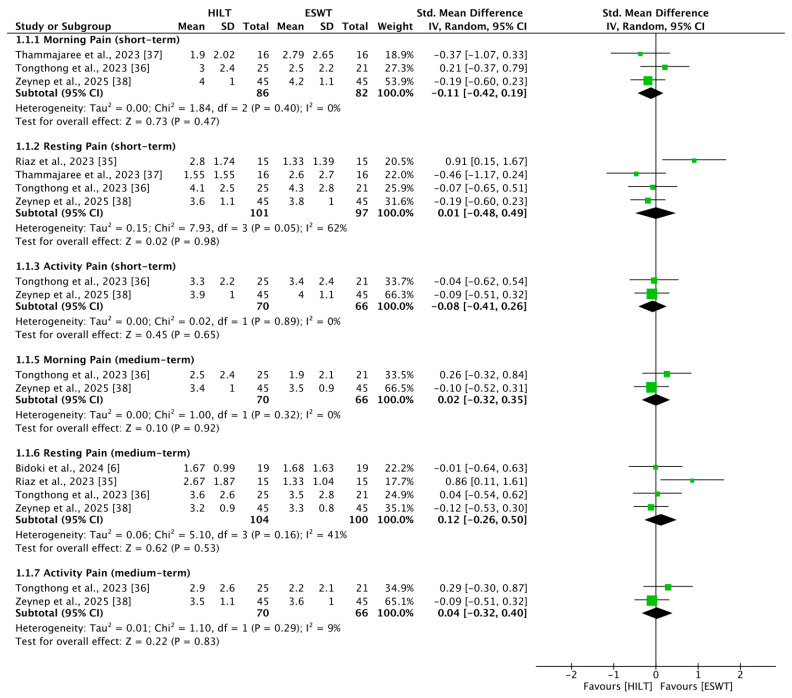
Forest plots of pain [6,35,36,37,38]. CI, confidence interval; ESWT, extracorporeal shockwave therapy; HILT, high-intensity laser therapy; SD, standard deviation.

Three studies [36,37,38] reported outcomes related to short-term morning pain, including 86 patients in the HILT group and 82 in the ESWT group. The heterogeneity among studies was low (I^2^ = 0%, *p* = 0.40). There was no statistically significant difference in short-term morning pain between the HILT and ESWT groups (SMD = −0.11; 95% CI: −0.42 to 0.19) (Figure 2).

### 3.5. Short-Term Resting Pain

Four studies [35,36,37,38] reported outcomes related to short-term resting pain, including 101 patients in the HILT group and 97 in the ESWT group. The heterogeneity among studies was moderate (I^2^ = 62%, *p* = 0.05). No statistically significant difference in short-term resting pain was observed between the HILT and ESWT groups (SMD = 0.01; 95% CI: −0.48 to 0.49) (Figure 2).

### 3.6. Short-Term Activity Pain

Two studies [36,38] reported outcomes related to short-term activity pain, including 70 patients in the HILT group and 66 in the ESWT group. Heterogeneity between the studies was low (I^2^ = 0%, *p* = 0.89). There was no statistically significant difference in short-term activity pain between the HILT and ESWT groups (SMD = −0.08; 95% CI: −0.41 to 0.26) (Figure 2).

### 3.7. Medium-Term Morning Pain

Two studies [36,38] reported outcomes related to medium-term morning pain, including 70 patients in the HILT group and 66 in the ESWT group. Heterogeneity between the studies was low (I^2^ = 0%, *p* = 0.32). No statistically significant difference in medium-term morning pain was observed between the HILT and ESWT groups (SMD = 0.02; 95% CI: −0.32 to 0.35) (Figure 2).

### 3.8. Medium-Term Resting Pain

Four studies [6,35,36,38] reported outcomes related to medium-term resting pain, including 104 patients in the HILT group and 100 in the ESWT group. Heterogeneity among studies was moderate (I^2^ = 41%, *p* = 0.16). No statistically significant difference in medium-term resting pain was observed between the HILT and ESWT groups (SMD = 0.12; 95% CI: −0.26 to 0.50) (Figure 2).

### 3.9. Medium-Term Activity Pain

Two studies [36,38] reported outcomes related to medium-term activity pain, including 70 patients in the HILT group and 66 in the ESWT group. Heterogeneity between the studies was low (I^2^ = 9%, *p* = 0.29). No statistically significant difference in medium-term activity pain was observed between the HILT and ESWT groups (SMD = 0.04; 95% CI: −0.32 to 0.40) (Figure 2).

### 3.10. Short-Term Foot Function

**Figure 3:**
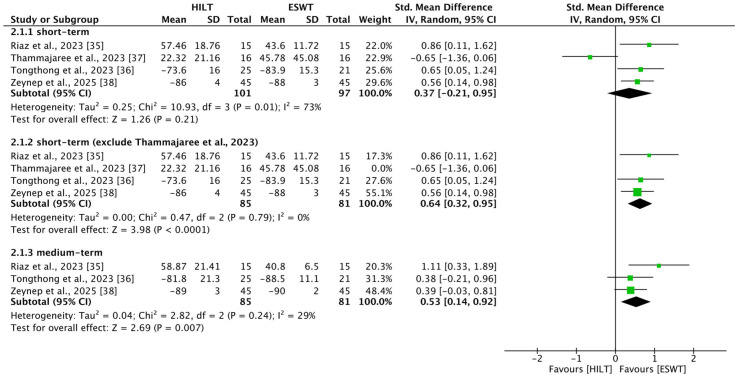
Forest plots of foot function [35,36,37,38]. CI, confidence interval; ESWT, extracorporeal shockwave therapy; HILT, high-intensity laser therapy; SD, standard deviation.

Four studies [35,36,37,38] reported outcomes related to short-term foot function, including 101 patients in the HILT group and 97 in the ESWT group. Heterogeneity among studies was high (I^2^ = 73%, *p* = 0.01). No statistically significant difference in short-term foot function was observed between the HILT and ESWT groups (SMD = 0.37; 95% CI: −0.22 to 0.95) (Figure 3).

Owing to high heterogeneity, the study by Thammajaree et al. was excluded from a sensitivity analysis because of significant differences in baseline characteristics [37]. After excluding this study, three studies [35,36,38] reported outcomes related to short-term foot function, including 85 patients in the HILT group and 81 in the ESWT group. Heterogeneity decreased substantially (I^2^ = 0%, *p* = 0.79), and a statistically significant difference in short-term foot function emerged in favor of the ESWT group (SMD = 0.64; 95% CI: 0.32 to 0.95) (Figure 3).

### 3.11. Medium-Term Foot Function

Three studies [35,36,38] reported outcomes related to medium-term foot function, including 85 patients in the HILT group and 81 in the ESWT group. Heterogeneity among studies was low (I^2^ = 29%, *p* = 0.24). A statistically significant difference in medium-term foot function was observed in favor of the ESWT group (SMD = 0.53; 95% CI: 0.14 to 0.92) (Figure 3).

### 3.12. GRADE Assessment

The quality of evidence was evaluated using the GRADE approach. Overall, the quality ranged from moderate to low, likely due to risks of bias and heterogeneity among the included studies. Details of the quality assessment are presented in Table 5.

## 4. Discussion

### 4.1. Summary of Main Findings

In this systematic review, the comparative effects of ESWT and HILT on functional improvement and pain relief in patients with plantar fasciitis were examined. After excluding the study by Thammajaree et al. [37] owing to high heterogeneity in the baseline characteristics of foot function, moderate-quality evidence revealed that ESWT was significantly more effective than HILT in improving foot function in both the short-term (<1 month) and medium-term (1–3 months). In terms of pain outcomes, the findings were more variable. Low-quality evidence was associated with short-term resting pain, but no significant difference was found between the two groups. On the other hand, moderate-quality evidence indicated no statistically significant differences between the two interventions in terms of short-term morning pain, short-term activity pain, medium-term morning pain, medium-term resting pain, or medium-term activity pain.

Prior meta-analyses generally support ESWT for plantar fasciitis, though effects are often imprecise due to protocol heterogeneity and ESWT outcomes may depend on treatment parameters [3,13]. HILT meta-analyses also report functional improvement but with limited trials and heterogeneous protocols [39], and a broader ESWT-versus-laser meta-analysis found no clear functional superiority of ESWT over HILT with low to very-low certainty [40]. In this context, our head-to-head synthesis suggests a modest short- to medium-term functional advantage of ESWT over HILT (after harmonizing scale direction), while pain outcomes appear largely comparable.

### 4.2. Evidence-Based Perspectives on ESWT and HILT Modalities

ESWT relieves PF-related pain by stimulating nerve receptors, reducing nerve sensitivity and conduction, altering nociceptor activity, promoting neovascularization, and enhancing collagen synthesis in degenerative tissues [13]. Previous studies have shown that ESWT results in better pain relief and higher success rates than other conservative PF treatments do [2,12]. A 2002 systematic review by Ogden et al., which included 20 studies, recommended ESWT as a treatment option prior to considering surgery [41]. The authors also noted that ESWT may be more favorable than corticosteroid injections, which carry risks such as plantar fascia rupture and lack sufficient long-term safety data, especially regarding repeated use [41]. Similarly, a 2020 meta-analysis by Sun et al. concluded that ESWT offered better outcomes in terms of pain reduction, success rate, time to return to work, and fewer complications than other therapies, such as placebo, ultrasound, and local physical therapy [42].

Compared with radial ESWT, focused ESWT penetrates deeper into tissue because of concentrated energy delivery. Radial ESWT spreads the energy across a wider surface area and disperses it more superficially. Although both forms are used in PF treatment, there is no consensus on which is more effective. Sun et al.’s meta-analysis, which included 9 studies and 935 patients, showed that both types could relieve chronic PF pain but did not establish superiority due to study heterogeneity [42]. Another meta-analysis by Wang et al. in 2019, which analyzed 14 RCTs, suggested that focused ESWT might be more effective at medium energy levels (energy flux density: 0.10–0.20 mJ/mm^2^) [13]. However, the limited number of studies and variability in protocols weaken these conclusions. In contrast, a network meta-analysis by Chang et al. reported that radial ESWT was the most effective option [14]. The authors noted that the limited number of included studies and heterogeneity led to inconsistent results. In this review, four studies applied radial ESWT as treatment [6,36,37,38], whereas the other study did not specify the type of ESWT used [35].

In addition to ESWT monotherapy, some studies have investigated the efficacy of combining ESWT with plantar fascia-specific stretching protocols. A 2010 randomized controlled trial by Rompe et al. [43] compared plantar fascia-specific stretching alone with low-energy radial ESWT in patients with acute plantar fasciopathy (≤6 weeks). Their findings indicated that stretching exercises produced significantly better improvements in pain and function at two and four months, with higher patient satisfaction than ESWT did (*p* < 0.01), although the differences diminished at the 15-month follow-up. In contrast, in a 2015 follow-up study [44], the same research group evaluated 152 patients with chronic plantar fasciopathy (≥12 months) and reported that a combined protocol of radial ESWT plus stretching was significantly more effective than ESWT alone at two and four months (*p* < 0.01), although the benefit did not persist at 24 months. These findings suggest that the clinical stage may play a critical role in determining the most effective treatment strategy and that a multimodal approach may enhance treatment efficacy.

Laser therapy, including HILT and low-level laser therapy (LLLT), provides a noninvasive and painless option for managing musculoskeletal disorders. Both operate in the near-infrared range; LLLT commonly uses wavelengths of 830, 850, or 950 nm, and HILT typically uses 1064 nm. Despite this difference, their tissue penetration depths are similar, primarily affecting superficial layers [16,45]. Compared with LLLT, HILT delivers more power (above 0.5 W), which allows for greater coverage and induces photothermal effects. HILT has been reported to increase metabolism, promote beta-endorphin release, and exert anti-inflammatory, anti-edema, and analgesic effects. The application of HILT in many musculoskeletal diseases, including PF, has been proven effective, with favorable results in terms of pain reduction and functional regain. A randomized controlled trial conducted by Naruseviciute et al. [46] involving 102 patients with unilateral plantar fasciitis compared HILT and LLLT over an eight-session, three-week treatment protocol with follow-up at four weeks. The trial found no statistically significant differences between the two groups in terms of pain reduction, pain threshold, or plantar fascia thickness. However, a significantly greater proportion of participants in the HILT group reported treatment efficacy above 50%, suggesting higher patient-perceived benefit despite comparable objective outcomes [46].

### 4.3. Clinical Trials Comparing HILT and ESWT

Thammajaree et al. [37] conducted a randomized clinical trial and revealed that either radial ESWT or HILT can alleviate pain. Notably, increased skin blood flow was found in the radial ESWT group after three weeks of treatment, likely due to the neovascularization effect of ESWT. Moreover, a reduction in plantar fascia thickness was observed in both the radial ESWT and HILT groups. This novel finding suggests that the tissue-healing effects promoted by HILT may achieve comparable outcomes in reducing plantar fascia thickness to those of ESWT. Karakuzu Güngör et al. [38] evaluated the effectiveness of ESWT and HILT in managing calcaneal spur-related symptoms. The evaluation of range of motion (ROM) revealed marked improvements in both groups from baseline to 3 months after intervention, with continued progress observed at the 3-month follow-up. Both ESWT and HILT significantly increased flexion, abduction, internal rotation, and external rotation.

Hassan et al. [40] conducted a systematic review and meta-analysis that included multiple musculoskeletal disorders, with plantar fasciitis as one of the targeted conditions. They incorporated both HILT and LLLT as comparators to ESWT and evaluated outcomes such as strength, range of motion, and quality of life. Furthermore, they observed a marginal functional advantage of ESWT over LLLT but not over HILT. However, their findings were limited by substantial clinical and statistical heterogeneity, inconsistent reporting of treatment protocols, and a generally low to very low quality of evidence due to risk of bias and imprecision across studies.

Despite these mixed outcomes, much of the inconsistency can be attributed to differences in outcome instruments, follow-up durations, and protocol design rather than true contradictions. Some trials relied on the FFI, where higher scores reflect greater disability, whereas others used the FAAM, where higher scores indicate better function, complicating cross-study comparisons. The AOFAS score combines patient-reported and clinician-assessed items, introducing subjectivity and further limiting validity. Follow-up periods also varied, with HILT sometimes demonstrating more rapid short-term pain relief, whereas ESWT showed more sustained functional benefits in the medium-term. Protocol variability in both laser parameters and ESWT energy dosing likely contributed further heterogeneity. Accordingly, while individual studies reported mixed findings, our pooled meta-analysis indicated no significant difference in pain reduction but demonstrated a moderate short- to medium-term advantage of ESWT in functional improvement.

### 4.4. Treatment Heterogeneity and Standardization

One of the major challenges identified in this review was the substantial heterogeneity in treatment parameters among the studies we included. In these studies, there was no consistent standard for ESWT or HILT in terms of energy settings, treatment duration, number of sessions, or application intervals. The observed moderate-to-high heterogeneity in certain outcomes, such as short-term resting pain and foot function, likely stems from several clinical and methodological factors. First, treatment protocols varied substantially; most included trials did not report exact session intervals and instead described schedules only as the total number of sessions delivered over a specified number of weeks. In addition, intervention dosing differed markedly, with HILT energy ranging from 720 to 90,000 J per session and ESWT delivering 2000 to 6000 shocks per session. Second, the use of co-interventions—such as stretching programs, insoles, and orthoses—was inconsistent across studies, which may have confounded the isolated effects of the primary modalities. Lastly, differences in patient characteristics and the chronicity of plantar fasciitis could also contribute to the variability in therapeutic response. Our interpretation that protocol variability may contribute to heterogeneity is consistent with a recent meta-analysis/meta-regression showing that ESWT effectiveness and tolerability can be parameter-dependent, underscoring the importance of dosing considerations when comparing ESWT with HILT [47]. This lack of standardization likely contributed to variations in outcomes and complicated the interpretation of comparative efficacy. There is currently no universally accepted dosing guideline for HILT. While the World Association for Laser Therapy (WALT) [48] provides standardized protocols, these apply primarily to LLLT, and not to HILT. In contrast, the International Society for Medical Shockwave Treatment (ISMST) [49] has published evidence-based treatment recommendations for ESWT across various musculoskeletal conditions, including plantar fasciitis. According to ISMST guidelines, radial ESWT for plantar fasciitis should be delivered at a pressure strength of 2–4 bar, frequency up to 10 Hz, and 2000–3000 pulses per session. Among the studies included in our review, only one RCT [36] strictly adhered to the ISMST-recommended protocol for plantar fasciitis. The remaining studies exhibited variations in dosing and application protocols, potentially contributing to statistical heterogeneity and bias in the pooled outcomes.

When treatment heterogeneity was analyzed more closely, the total delivered energy of HILT emerged as a potential factor influencing comparative outcomes. For instance, Riaz et al. [35] administered the highest cumulative HILT dose (90,000 J) and found ESWT to be superior in both pain and function. Zeynep et al. [38] delivered the second highest energy (41,960 J) and reported that pain reduction favored HILT while functional improvement favored ESWT. At an intermediate energy level, Tongthong et al. [36] (11,529 J) observed no differences between the modalities, whereas low-dose protocols yielded different patterns: Thammajaree et al. [37] (900 J) favored HILT for both outcomes, while Bidoki et al. [6] (720 J) found no significant differences. These findings suggest that total dosage may shape the relative performance of ESWT and HILT, though the direction of effect is not linear.

This observation aligns with broader systematic reviews. A systematic review and meta-analysis conducted by Arroyo-Fernández et al. [50] noted that HILT effectiveness on musculoskeletal pain did not differ significantly across predefined dosage subgroups (≤10 J/cm^2^, 10–50 J/cm^2^, 50–100 J/cm^2^, 100–300 J/cm^2^), but the treatment effect tended to diminish at higher dosages, raising questions about whether excessively high energy could attenuate clinical gains. In contrast, de la Barra Ortiz et al. [39] recommended standardized parameters for plantar fasciitis, including a wavelength of 1064 nm, 12 W continuous mode, an energy density of at least 120 J/cm^2^, and a cumulative total energy of no less than 3000 J for an area of 25 cm^2^, delivered across 8–10 sessions over three weeks. Taken together, these findings highlight that variability in HILT dosage protocols not only contributes to heterogeneity across trials but may also partially explain the divergent comparative outcomes against ESWT. Future research should therefore prioritize protocol standardization and dose–response exploration to clarify whether there is an optimal therapeutic window for HILT in plantar fasciitis.

### 4.5. Clinical Implications

To our knowledge, few systematic reviews have directly compared the effects of ESWT and HILT in the treatment of PF. On the basis of our meta-analytic findings, ESWT demonstrated statistically superior outcomes in terms of improved foot function compared with HILT in both the short-and medium-term. In practice, patients with plantar fasciitis often prioritize pain reduction, particularly relief from morning or activity-related pain. Since both interventions appear comparable in terms of pain-related outcomes, factors such as treatment tolerability, patient preference, and side effects should guide therapeutic choices. ESWT may cause discomfort, such as needle-like sensations, during treatment, and approximately 20% of patients experience side effects, including temporary skin redness and ecchymosis [15]. Therefore, clinicians should thoroughly inform patients of these potential adverse effects before initiating ESWT to ensure shared decision-making in clinical practice. In our study, three RCTs [6,35,36] incorporated adjunctive treatments, including stretching exercises, insoles, self-care and footwear education, postural correction, and analgesics, into their protocols to enhance overall therapeutic outcomes. For patients whose primary concern is pain, adjunctive options such as plantar fascia-specific stretching and anti-inflammatory modalities may be considered [2,43]. In chronic cases, combining ESWT with stretching protocols has shown improved outcomes, particularly in terms of functional recovery [44]. These findings indicate that personalized and multimodal strategies may offer enhanced benefits over monotherapy alone.

### 4.6. Future Research Directions

Future studies should adopt standardized treatment protocols. For HILT, the current absence of universally accepted dosing guidelines highlights the need to establish evidence-based recommendations tailored specifically to this modality. For ESWT, adherence to established ISMST protocols is crucial, as these provide disease-specific parameters that minimize methodological variability. Additionally, incorporating objective outcome measures, such as ultrasonographic assessments of plantar fascia thickness, changes in skin blood flow, and ROM, would allow for a more comprehensive evaluation of both structural and functional changes induced by each intervention. These biomarkers could also facilitate the identification of potential responder profiles, thereby informing more personalized treatment strategies.

### 4.7. Study Strengths and Limitations

This review is among the few reviews to directly assess these two modalities in patients with plantar fasciitis using a meta-analytic framework. Unlike previous reviews, such as that of de la Barra Ortiz et al. [39], which focused solely on the analgesic and Foot and Ankle Outcome Score effects of HILT compared with placebo or alternative therapies, this study offers a direct comparison between HILT and ESWT.

However, several limitations should be acknowledged in this review. First, the innovative nature of our research question limited the availability of prior systematic reviews or meta-analyses for comparison. As one of the first studies to address this comparison, our findings should be interpreted as preliminary. Second, the small number of randomized controlled trials directly comparing the two modalities restricted the strength of the evidence, restricted subgroup analyses, and raised concerns about potential publication bias. Additionally, our search strategy primarily focused on peer-reviewed literature in major databases; therefore, the exclusion of unpublished studies or gray literature may have introduced a potential for publication bias. Furthermore, substantial heterogeneity existed across studies in terms of treatment protocols, patient populations, follow-up durations, and outcome measures. The lack of standardized ultrasonographic data further constrains the comprehensive evaluation of tissue-level changes. In addition, our risk of bias assessment revealed that participant and therapist blinding was largely absent across most studies, likely owing to the inherent characteristics of the interventions. This methodological limitation potentially introduces performance bias, necessitating careful interpretation of the study findings.

## 5. Conclusions

While the pooled results suggested a trend toward greater functional improvement with ESWT than with HILT in the short-and medium-term, the effect sizes were small. No significant between-group differences were observed in pain-related outcomes. Despite suggesting potential benefits of both modalities, the comparative superiority of ESWT versus HILT cannot be confirmed due to methodological limitations, including the small number of studies and moderate-to-high heterogeneity. This heterogeneity likely reflects substantial protocol differences, inconsistent co-interventions, and variability in patient characteristics and disease chronicity. The constraints on statistical power due to small sample sizes mean that these findings should be interpreted with caution. Further high-quality, large-scale randomized controlled trials with standardized methodologies are needed to better inform clinical decision-making.

## Figures and Tables

**Table 1:** Characteristics of the included studies.

Author, Year	Country		Intervention Groups						Post Assessment	Followed up Timing	Outcome Measurement	Adverse Event(s)
			Number of Patients	Age (y), Mean (SD)	Sex Distribution	Dropouts	Intervention Protocol	Total Dose				
Bidoki et al., 2024 [6]	Iran	Experimental group	19	44.26 (9.53)	Male: 11 (57.9%) Female: 8 (42.1%)	6 out of 25	HILT was performed using an Nd:YAG laser (GaIA’S, GIGAA LASER, VELASII-30B, UK) 980 ± 10 nm, applied to the plantar fascia at 30 W, 8 J/cm^2^, with a 10 cm^2^ spot beam diameter. Patients also received stretching exercises and insoles (if needed).	720 J	-	3 months after the intervention	VAS, HTI Scores, SF36 Scores	no
		Control group	19	45.05 (6.85)	Male: 16 (84.2%) Female: 3 (15.8%)	6 out of 25	The ESWT was performed using a Master Plus MP100 (STORZ MEDICAL, Tägerwilen, Switzerland, Radial type) in low-energy mode with an R15 transmitter (2–3 Bar, 3000 pulses, 12 MHz) followed by a D20-S transmitter (1.8–3 Bar, 3000 pulses, 15 MHz). Patients also received stretching exercises and insoles (if needed).	54,000 shocks				Bruising (*n* = 1)
Thammajaree et al., 2023 [37]	Thailand	Experimental group	16	46.06 (8.55)	Male: 8 (50.0%) Female: 8 (50.0%)	0 out of 16	HILT was performed using an SH1 (ASA Srl, Arcugnano, Italy) for six sessions over three weeks (twice weekly) with a pulsed Nd:YAG laser (1064 nm, 6 W, 5 J/cm^2^, 250 µs, 5 mm probe). Treatment included three phases: (1) fast scanning along the plantar aponeurosis (60 J, 2 min), (2) targeted application on six painful spots (30 J total), and (3) slow scanning on the plantar aponeurosis (60 J, 2 min). The total energy per session was 150 J (~5 min). During treatment, patients were prone with ankles at the edge of the couch, wearing safety goggles.	900 J	3 weeks after the intervention	-	VAS, Foot Function Index, Plantar fascia thickness, Skin blood flow	no
		Control group	16	48.12 (11.96)	Male: 8 (50.0%) Female: 8 (50.0%)	0 out of 16	The radial type ESWT was performed using a BTL-6000 SWT (BTL Corporate, Stevenage, UK) at 2–3 Bar, 10 Hz, 2000 shocks per session for six sessions (twice weekly). During treatment, patients were prone with the ankle at the edge of the couch. The therapist identified the most painful spot and applied 500 shocks around it, 1000 shocks in a circular motion at the spot, and 500 shocks along the plantar aponeurosis.	12,000 shocks				no
Tongthong et al., 2023 [36]	Thailand	Experimental group	25	51.2 (14.9)	Male: 11 (44.0%) Female: 14 (56.0%)	1 out of 26	The HILT group received pulsed laser treatment using an HIRO 3 (ASA, Arcugnano, Italy) with a pulsed Nd:YAG laser (1064 nm, 3 kW peak power, 10.5 W mean power, 10–40 Hz, 0.1% duty cycle, 0.5 cm probe, 0.2 cm^2^ spot size). The modified protocol included 9 sessions (3 per week for 3 weeks). Treatment followed a three-phase regimen on the plantar fascia, delivering a total of 1281.1 J per session: (1) initial phase (624 J), (2) targeted phase (33.1 J), and (3) manual scanning phase (624 J). Laser fluency ranged from 360 to 1780 mJ/cm^2^ with pulse durations of 120–150 µs. All patients received education on self-care and footwear, a plantar fascia stretching program (twice daily), and were allowed acetaminophen as needed, with adherence logged.	11,529.9 J	3 weeks after the intervention	7 weeks after the intervention	VAS, foot and ankle ability measure	no
		Control group	21	47.5 (13.2)	Male: 4 (19.0%) Female: 17 (81.0%)	5 out of 26	The radial type ESWT protocol followed the Yinilmez study, with one session per week for three weeks (three sessions total) using a Duolith^®^ SD1 (Storz Medical, Tägerwilen, Switzerland). Treatment was applied with an R15 (15 mm) transmitter at 0.38 mJ/mm^2^ (40 mm depth), 2 Bar, 2000 shocks/min at 10 Hz, 1000 shocks were delivered at the plantar fascia insertion and 1000 shocks along the fascia in a circular motion. All patients received education on self-care and footwear, a plantar fascia stretching program (twice daily), and were allowed acetaminophen as needed, with adherence logged.	6000 shocks				no
Riaz et al., 2023 [35]	Pakistan	Experimental group	15	38.06 (12.64)	Not reported	Not reported	Diowave 60 W Class IV Laser therapy was applied at 980 nm, 30 W, 10,000 J per session using the scanning method. Treatment was performed three times per week for three weeks (9 sessions total). All patients were given some exercises to follow for the home plan and provided some postural correction guidelines.	90,000 J	3 weeks after the intervention	2 months after the intervention	VAS, Foot Function Index	no
		Control group	15	39.66 (10.05)	Not reported	Not reported	BTL-6000 shockwave therapy (unknown type ESWT) was applied at 2.0 Bar, 10 Hz, 2000 shocks per session. Treatment was delivered twice a week for three weeks, with 1000 shocks at the heel (fascia insertion) and 1000 shocks along the fascia in a circular manner. All patients were given some exercises to follow for the home plan and provided some postural correction guidelines.	12,000 shocks				no
Zeynep et al., 2025 [38]	Turkey	Experimental group	45	47.33 (7.87)	Male: 22 (48.9%) Female: 23 (51.1%)	Not reported	HILT group using a BTL-6000^®^ device (BTL Corporate, Stevenage, UK). Two modes were applied: 10 W/12 J/cm^2^ (analgesic dose) for 2 min, and 7 W/120 J/cm^2^ (biostimulation dose) for 7 min and 8 s. Treatment was applied five times per week, for a total of 10 sessions over two weeks.	41,960 J	HILT group: 2 weeks after the intervention; ESWT group: 3 weeks after the intervention	3 months after the intervention	VAS, Roles and Maudsley scores, AOFAS scale	no
		Control group	45	48.77 (9.64)	Male: 15 (33.3%) Female: 30 (66.7%)	Not reported	ESWT group using a radial-type shock wave device (Masterplus MP200 Elite– Storz Medical AG, Kreuzlingen, Switzerland). Treatment parameters were set to a frequency of 12–15 Hz, 2–3 bar pressure, and 2500 pulses per session. Therapy was administered every three days, for a total of five sessions over three weeks. Treatment targeted the medial calcaneal area and the most painful points identified through palpation	12,500 shocks				no

AOFAS scale, American Orthopaedic Foot and Ankle Society scale; ESWT, extracorporeal shockwave therapy; HILT, high-intensity laser therapy; HTI: Heel tenderness index; SD, standard deviation; SF36: Short Form-36 Health Survey; VAS: Visual analog scale.

**Table 2:** High-intensity laser therapy parameters.

	Treatment Protocol	Mode	Power (W)	Average Power (W)	Fluence (J/cm^2^)	Energy Dose (J)/Session	Spot Diameter (mm)
Bidoki et al., 2024 [6]	9 sessions over 3 weeks	Not reported	30	Not reported	8	80	35.6
Thammajaree et al., 2023 [37]	6 sessions over 3 weeks	pulsed	0.5	0.5	5	150	5
Tongthong et al., 2023 [36]	9 sessions over 3 weeks	pulsed	Not reported	10.5	0.36–1.78	1281.1	5
Riaz et al., 2023 [35]	9 sessions over 3 weeks	Not reported	30	Not reported	Not reported	10,000	Not reported
Zeynep et al., 2025 [38]	10 sessions over 2 weeks	Not reported	7–10	7.65	12–120	4196	Not reported

**Table 3:** Extracorporeal shockwave therapy parameters.

	Treatment Protocol	ESWT Type	Energy Density	Frequency
Bidoki et al., 2024 [6]	6000 shocks each session 9 sessions over 3 weeks	Radial	R15 transmitter: 2–3 Bar D20-S transmitter: 1.8–3 Bar	R15 transmitter: 12 MHz D20-S transmitter: 15 MHz
Thammajaree et al., 2023 [37]	2000 shocks each session 6 sessions over 3 weeks	Radial	2–3 Bar	10 Hz
Tongthong et al., 2023 [36]	2000 shocks each session 3 sessions over 3 weeks	Radial	0.38 mJ/mm^2^, 2 Bar	10 Hz
Riaz et al., 2023 [35]	2000 shocks each session 6 sessions over 3 weeks	Not reported	2.0 Bar	10 Hz
Zeynep et al., 2025 [38]	2500 shocks each session 5 sessions over 3 weeks	Radial	2–3 Bar	12–15 Hz

**Table 4:** PEDro scale.

	1 *	2	3	4	5	6	7	8	9	10	11	Total
Bidoki et al., 2024 [6]	Y	Y	N	Y	N	N	Y	Y	Y	Y	Y	7
Thammajaree et al., 2023 [37]	Y	Y	N	Y	N	N	Y	Y	Y	Y	Y	7
Tongthong et al., 2023 [36]	Y	Y	N	Y	N	N	Y	Y	Y	Y	Y	7
Riaz et al., 2023 [35]	Y	Y	N	Y	N	N	Y	Y	Y	Y	Y	7
Zeynep et al., 2025 [38]	Y	Y	N	Y	N	N	N	Y	Y	Y	Y	6

PEDro scale criteria: 1, eligibility criteria and source of participants; 2, random allocation; 3, concealed allocation; 4, baseline comparability; 5, blinded participants; 6, blinded therapists; 7, blind assessors; 8, adequate follow-up; 9, intention-to-treat analysis; 10, between-group comparisons; 11, point estimates and variability. * Not included in the calculation of the total score; N: no; Y: yes.

**Table 5:** GRADE assessment.

Certainty Assessment							Number of Patients		Effect	Certainty	Importance
Number of Studies	Study Design	Risk of Bias	Inconsistency	Indirectness	Imprecision	Other Considerations	Experimental Group	Control Group	Absolute (95% CI)		
Morning Pain (short-term)											
3	randomized controlled trials	serious ^a^	not serious	not serious	not serious	none	86	82	SMD 0.11 SD higher (0.19 lower to 0.42 higher)	⨁⨁⨁◯ Moderate ^a^	Important
Resting Pain (short-term)											
4	randomized controlled trials	serious ^a^	serious ^b^	not serious	not serious	none	101	97	SMD 0.01SD higher (0.48 lower to 0.49 higher)	⨁⨁◯◯ Low ^a,b^	Important
Activity Pain (short-term)											
2	randomized controlled trials	serious ^a^	not serious	not serious	not serious	none	70	66	SMD 0.08 SD higher (0.26 lower to 0.41 higher)	⨁⨁⨁◯ Moderate ^a^	Important
Morning Pain (medium-term)											
2	randomized controlled trials	serious ^a^	not serious	not serious	not serious	none	70	66	SMD 0.02 SD higher (0.32 lower to 0.35 higher)	⨁⨁⨁◯ Moderate ^a^	Important
Resting Pain (medium-term)											
4	randomized controlled trials	serious ^a^	not serious	not serious	not serious	none	104	100	SMD 0.12 SD higher (0.26 lower to 0.50 higher)	⨁⨁⨁◯ Moderate ^a^	Important
Activity Pain (medium-term)											
2	randomized controlled trials	serious ^a^	not serious	not serious	not serious	none	70	66	SMD 0.04 SD higher (0.32 lower to 0.40 higher)	⨁⨁⨁◯ Moderate ^a^	Important
Foot Function (short-term)											
4	randomized controlled trials	serious ^a^	serious ^c^	not serious	not serious	none	101	97	SMD 0.37 SD higher (0.21 lower to 0.95 higher)	⨁⨁◯◯ Low ^a,c^	Important
Foot Function (short-term, excluding Thammajaree et al., 2023 [37])											
3	randomized controlled trials	serious ^a^	not serious	not serious	not serious	none	85	81	SMD 0.64 SD higher (0.32 lower to 0.95 higher)	⨁⨁⨁◯ Moderate ^a^	Important
Foot Function (medium-term)											
3	randomized controlled trials	serious ^a^	not serious	not serious	not serious	none	85	81	SMD 0.53 higher (0.14 lower to 0.92 higher)	⨁⨁⨁◯ Moderate ^a^	Important

CI: confidence interval; SMD: standardized mean difference. Explanations: ^a^. Serious risk of bias: The evidence was downgraded due to a lack of blinding among participants and therapists, which is inherent to the nature of the interventions (HILT and ESWT). ^b^. Serious inconsistency: The evidence was downgraded due to moderate statistical heterogeneity, suggesting variability in treatment effects across the included studies. ^c^. Serious inconsistency: The evidence was downgraded due to high statistical heterogeneity, likely resulting from differences in baseline characteristics or treatment protocols. Certainty of evidence (GRADE): ⊕⊕⊕⊕ = High certainty; ⊕⊕⊕◯ = Moderate certainty; ⊕⊕◯◯ = Low certainty; ⊕◯◯◯ = Very low certainty. Filled circles (⊕) indicate the number of GRADE levels met, whereas open circles (◯) indicate downgrading due to limitations in the evidence.

## Data Availability

All the data generated or analyzed in this study are included in this published article.

## Abbreviations

The following abbreviations are used in this manuscript:

ADL	Activities of Daily Living
AOFAS	American Orthopaedic Foot & Ankle Society
CI	Confidence Interval
ESWT	Extracorporeal Shockwave Therapy
FAAM	Foot and Ankle Ability Measure
FFI	Foot Function Index
GRADE	Grading of Recommendations Assessment, Development and Evaluation
HILT	High-Intensity Laser Therapy
HTI	Heel Tenderness Index
PEDro	Physiotherapy Evidence Database
PF	Plantar Fasciitis
PPT	Pressure Pain Threshold
PRISMA	Preferred Reporting Items for Systematic Reviews and Meta-Analyses
RCT	Randomized Controlled Trial
ROM	Range of Motion
SMD	Standardized Mean Difference
USG	Ultrasonography

## Appendix A

Keywords used for identifying relevant articles in various electronic databases.

**Database-1**	**Search Terms**
PubMed (inception: 1782)	Search for articles published from database inception to 13 July 2025.
#1	Shockwave
#2	Shock wave
#3	ESWT
#4	Extracorporeal Shockwave Therapy [MeSH]
#5	#1 OR #2 OR #3 OR #4
#6	laser
#7	Laser therapy [MeSH]
#8	#6 OR #7
#9	plantar
#10	heel
#11	Fasciitis, Plantar [MeSH]
#12	#9 OR #10 OR #11
#13	#5 AND #8 AND #12
Filter	Randomized controlled trial
Note: # indicates the sequential search set number used in the search strategy; Boolean operators (OR/AND) were applied to combine search sets.	

**Database-2**	**Search Terms**
EMBASE (inception: 1947)	Search for articles published from database inception to 13 July 2025.
#1	Shockwave
#2	Shock wave
#3	ESWT
#4	Extracorporeal Shockwave Therapy/exp
#5	#1 OR #2 OR #3 OR #4
#6	laser
#7	Laser therapy/exp
#8	#6 OR #7
#9	plantar
#10	heel
#11	Fasciitis, Plantar/exp
#12	#9 OR #10 OR #11
#13	#5 AND #8 AND #12
Filter	Randomized controlled trial
Note: # indicates the sequential search set number used in the search strategy; Boolean operators (OR/AND) were applied to combine search sets.	

**Database-3**	**Search Terms**
Cochrane Library (inception: 1971)	Search for articles published from database inception to 13 July 2025.
#1	Shockwave
#2	Shock wave
#3	ESWT
#4	[Extracorporeal Shockwave Therapy] explode all trees
#5	#1 OR #2 OR #3 OR #4
#6	laser
#7	[Laser therapy] explode all trees
#8	#6 OR #7
#9	plantar
#10	heel
#11	[Fasciitis, Plantar] explode all trees
#12	#9 OR #10 OR #11
#13	#5 AND #8 AND #12
#14	Random *
#15	#13 AND #14
Note: # indicates the sequential search set number used in the search strategy; Boolean operators (OR/AND) were applied to combine search sets. Truncation using the asterisk (*) was applied in the search.	

**Database-4**	**Search Terms**
Scopus (inception: 1999)	Search for articles published from database inception to 13 July 2025.
#1	Extracorporeal Shockwave Therapy
#2	High intensity laser therapy
#3	Plantar fasciitis
#4	#1 AND #2 AND #3
Note: # indicates the sequential search set number used in the search strategy; Boolean operators (OR/AND) were applied to combine search sets.	

## References

[1] Lopes A.D., Hespanhol L.C., Yeung S.S., Costa L.O. (2012). What are the main running-related musculoskeletal injuries? A Systematic Review. Sports Med..

[2] Rhim H.C., Kwon J., Park J., Borg-Stein J., Tenforde A.S. (2021). A Systematic Review of Systematic Reviews on the Epidemiology, Evaluation, and Treatment of Plantar Fasciitis. Life.

[3] Babatunde O.O., Legha A., Littlewood C., Chesterton L.S., Thomas M.J., Menz H.B., van der Windt D., Roddy E. (2019). Comparative effectiveness of treatment options for plantar heel pain: A systematic review with network meta-analysis. Br. J. Sports Med..

[4] Klein S.E., Dale A.M., Hayes M.H., Johnson J.E., McCormick J.J., Racette B.A. (2012). Clinical presentation and self-reported patterns of pain and function in patients with plantar heel pain. Foot Ankle Int..

[5] Wearing S.C., Smeathers J.E., Urry S.R., Hennig E.M., Hills A.P. (2006). The pathomechanics of plantar fasciitis. Sports Med..

[6] Zare Bidoki M., Vafaeei Nasab M.R., Khatibi Aghda A. (2024). Comparison of High-intensity Laser Therapy with Extracorporeal Shock Wave Therapy in the Treatment of Patients with Plantar Fasciitis: A Double-blind Randomized Clinical Trial. Iran J. Med. Sci..

[7] Melo S.N.S., Ezekwesili A., Yurdi N.A., Murrell W.D., Maffulli N. (2020). Gold-Induced Cytokine (GOLDIC((R))) Injection Therapy in Patient with Plantar Fasciosis: A Case Report. Indian J. Orthop..

[8] Koc T.A., Bise C.G., Neville C., Carreira D., Martin R.L., McDonough C.M. (2023). Heel Pain-Plantar Fasciitis: Revision 2023. J. Orthop. Sports Phys. Ther..

[9] Oliva F., Piccirilli E., Tarantino U., Maffulli N. (2017). Percutaneous release of the plantar fascia. New surgical procedure. Muscles Ligaments Tendons J..

[10] Nery C., Raduan F., Mansur N., Baunfeld D., Del Buono A., Maffulli N. (2013). Endoscopic approach for plantar fasciopathy: A long-term retrospective study. Int. Orthop..

[11] Thomas J.L., Christensen J.C., Kravitz S.R., Mendicino R.W., Schuberth J.M., Vanore J.V., Weil L.S., Zlotoff H.J., Bouché R., Baker J. (2010). The diagnosis and treatment of heel pain: A clinical practice guideline-revision 2010. J. Foot Ankle Surg..

[12] Sun J., Gao F., Wang Y., Sun W., Jiang B., Li Z. (2017). Extracorporeal shock wave therapy is effective in treating chronic plantar fasciitis: A meta-analysis of RCTs. Medicine.

[13] Wang Y.C., Chen S.J., Huang P.J., Huang H.T., Cheng Y.M., Shih C.L. (2019). Efficacy of Different Energy Levels Used in Focused and Radial Extracorporeal Shockwave Therapy in the Treatment of Plantar Fasciitis: A Meta-Analysis of Randomized Placebo-Controlled Trials. J. Clin. Med..

[14] Chang K.V., Chen S.Y., Chen W.S., Tu Y.K., Chien K.L. (2012). Comparative effectiveness of focused shock wave therapy of different intensity levels and radial shock wave therapy for treating plantar fasciitis: A systematic review and network meta-analysis. Arch. Phys. Med. Rehabil..

[15] Roerdink R.L., Dietvorst M., van der Zwaard B., van der Worp H., Zwerver J. (2017). Complications of extracorporeal shockwave therapy in plantar fasciitis: Systematic review. Int. J. Surg..

[16] Elvir-Lazo O.L., White P.F., Yumul R. (2019). Cold laser therapy for acute and chronic pain management: A comparison of low-level and high-intensity laser therapy devices. Anesthesiol. News.

[17] Chow R.T., Johnson M.I., Lopes-Martins R.A., Bjordal J.M. (2009). Efficacy of low-level laser therapy in the management of neck pain: A systematic review and meta-analysis of randomised placebo or active-treatment controlled trials. Lancet.

[18] Pekyavas N.O., Baltaci G. (2016). Short-term effects of high-intensity laser therapy, manual therapy, and Kinesio taping in patients with subacromial impingement syndrome. Lasers Med. Sci..

[19] Li D., Zhang H., Chen B., Zhao Y.B., Wu W.J., Yuan Y., Ying Z.X. (2020). Experimental investigations on thermal effects of a long-pulse alexandrite laser on blood vessels and its comparison with pulsed dye and Nd:YAG lasers. Lasers Med. Sci..

[20] Song H.J., Seo H.J., Lee Y., Kim S.K. (2018). Effectiveness of high-intensity laser therapy in the treatment of musculoskeletal disorders: A systematic review and meta-analysis of randomized controlled trials. Medicine.

[21] Tkocz P., Matusz T., Kosowski Ł., Walewicz K., Argier Ł., Kuszewski M., Hagner-Derengowska M., Ptaszkowski K., Dymarek R., Taradaj J. (2021). A Randomised-Controlled Clinical Study Examining the Effect of High-Intensity Laser Therapy (HILT) on the Management of Painful Calcaneal Spur with Plantar Fasciitis. J. Clin. Med..

[22] Charles R., Fang L., Zhu R., Wang J. (2023). The effectiveness of shockwave therapy on patellar tendinopathy, Achilles tendinopathy, and plantar fasciitis: A systematic review and meta-analysis. Front. Immunol..

[23] Yadav S., Sharma S., Chatterjee S., Sharma A., Thakur S. (2025). Effect of LASER therapy on plantar fasciitis pain: Illuminating a promising treatment approach-a systematic review. Lasers Med. Sci..

[24] Higgins J.P.T., Thomas J., Chandler J., Cumpston M., Li T., Page M.J., Welch V.A. (2021). Cochrane Handbook for Systematic Reviews of Interventions.

[25] Moher D., Liberati A., Tetzlaff J., Altman D.G. (2009). Preferred reporting items for systematic reviews and meta-analyses: The PRISMA statement. Ann. Intern. Med..

[26] Delgado D.A., Lambert B.S., Boutris N., McCulloch P.C., Robbins A.B., Moreno M.R., Harris J.D. (2018). Validation of Digital Visual Analog Scale Pain Scoring With a Traditional Paper-based Visual Analog Scale in Adults. J. Am. Acad. Orthop. Surg. Glob. Res. Rev..

[27] Bovonsunthonchai S., Thong-On S., Vachalathiti R., Intiravoranont W., Suwannarat S., Smith R. (2020). Thai version of the foot function index: A cross-cultural adaptation with reliability and validity evaluation. BMC Sports Sci. Med. Rehabil..

[28] Van Lieshout E.M., De Boer A.S., Meuffels D.E., Den Hoed P.T., Van der Vlies C.H., Tuinebreijer W.E., Verhofstad M.H. (2017). American Orthopaedic Foot and Ankle Society (AOFAS) Ankle-Hindfoot Score: A study protocol for the translation and validation of the Dutch language version. BMJ Open.

[29] Ortega-Avila A.B., Sanchez-Morilla S., Galan-Hurtado M.H., Cervera-Garvi P., Garcia-Medina J., Marchena-Rodriguez A. (2025). Foot and Ankle Ability Measure (FAAM) Questionnaire: A Systematic Review. J. Am. Podiatr. Med. Assoc..

[30] de Morton N.A. (2009). The PEDro scale is a valid measure of the methodological quality of clinical trials: A demographic study. Aust. J. Physiother..

[31] Melsen W.G., Bootsma M.C., Rovers M.M., Bonten M.J. (2014). The effects of clinical and statistical heterogeneity on the predictive values of results from meta-analyses. Clin. Microbiol. Infect..

[32] Cohen J. (1988). Statistical Power Analysis for the Behavioral Sciences.

[33] Iorio A., Spencer F.A., Falavigna M., Alba C., Lang E., Burnand B., McGinn T., Hayden J., Williams K., Shea B. (2015). Use of GRADE for assessment of evidence about prognosis: Rating confidence in estimates of event rates in broad categories of patients. BMJ.

[34] Clarivate EndNote 20 (Desktop Software). https://endnote.com.

[35] Riaz S., Sattar A., Seemal P., Majeed R., Naveed A., Abid N., Bashir S. (2023). Comparison of Extracorporeal Shockwave and High-Intensity Laser in Treating Chronic Plantar Fasciitis-A single-blinded Randomized controlled trial. Pak. J. Med. Health Sci..

[36] Tongthong V., Boonyapracong T., Saipan P., Khanom K. (2024). Comparative Effectiveness of High-Intensity Laser Therapy and Radial Extracorporeal Shock Wave Therapy in Chronic Plantar Fasciitis: A Randomized, Single-Blind Clinical Trial. ASEAN J. Rehabil. Med..

[37] Thammajaree C., Theapthong M., Palee P., Pakpakorn P., Sitti T., Sakulsriprasert P., Bunprajun T., Thong-On S. (2023). Effects of radial extracorporeal shockwave therapy versus high intensity laser therapy in individuals with plantar fasciitis: A randomised clinical trial. Lasers Med. Sci..

[38] Karakuzu Gungor Z. (2025). Comparison of extracorporeal shock wave therapy and high-intensity laser therapy in the treatment of calcaneal spur-related symptoms: Clinical outcomes and functional improvement. J. Orthop. Surg. Res..

[39] de la Barra Ortiz H.A., Jélvez F., Parraguez D., Pérez F., Vargas C. (2023). Effectiveness of high-intensity laser therapy in patients with plantar fasciitis: A systematic review with meta-analysis of randomized clinical trials. Adv. Rehabil..

[40] Ismail Hassan M., Shafiek Mustafa Saleh M., Hesham Sallam M., Hesham Elkhodary H., Mohamed Sayed M., Samy H., Mohamed A.H., Ashour A.S., Mosaid E.M., Zaghloul M.H. (2025). Extracorporeal Shock Wave Therapy versus laser therapy in treating musculoskeletal disorders: A systematic review and meta-analysis. Lasers Med. Sci..

[41] Ogden J.A., Alvarez R.G., Marlow M. (2002). Shockwave therapy for chronic proximal plantar fasciitis: A meta-analysis. Foot Ankle Int..

[42] Sun K., Zhou H., Jiang W. (2020). Extracorporeal shock wave therapy versus other therapeutic methods for chronic plantar fasciitis. Foot Ankle Surg..

[43] Rompe J.D., Cacchio A., Weil L., Furia J.P., Haist J., Reiners V., Schmitz C., Maffulli N. (2010). Plantar fascia-specific stretching versus radial shock-wave therapy as initial treatment of plantar fasciopathy. J. Bone Jt. Surg. Am..

[44] Rompe J.D., Furia J., Cacchio A., Schmitz C., Maffulli N. (2015). Radial shock wave treatment alone is less efficient than radial shock wave treatment combined with tissue-specific plantar fascia-stretching in patients with chronic plantar heel pain. Int. J. Surg..

[45] Kaub L., Schmitz C. (2023). Comparison of the Penetration Depth of 905 nm and 1064 nm Laser Light in Surface Layers of Biological Tissue Ex Vivo. Biomedicines.

[46] Naruseviciute D., Kubilius R. (2020). The effect of high-intensity versus low-level laser therapy in the management of plantar fasciitis: Randomized participant blind controlled trial. Clin. Rehabil..

[47] Lippi L., Folli A., Moalli S., Turco A., Ammendolia A., de Sire A., Invernizzi M. (2024). Efficacy and tolerability of extracorporeal shock wave therapy in patients with plantar fasciopathy: A systematic review with meta-analysis and meta-regression. Eur. J. Phys. Rehabil. Med..

[48] World Association for Photobiomodulation Therapy WALT Recommendations. https://waltpbm.org/documentation-links/recommendations/.

[49] International Society for Medical Shockwave Treatment ISMST Guidelines. https://shockwavetherapy.org/ismst-guidelines/.

[50] Arroyo-Fernández R., Aceituno-Gómez J., Serrano-Muñoz D., Avendaño-Coy J. (2023). High-Intensity Laser Therapy for Musculoskeletal Disorders: A Systematic Review and Meta-Analysis of Randomized Clinical Trials. J. Clin. Med..

